# New-Type Shearing Interference Detection System Based on Double-Grating Structure for Suppressing the Skylight Background

**DOI:** 10.3390/s23104695

**Published:** 2023-05-12

**Authors:** Lei Dong, Bin Wang, Hongzhuang Li, Yuanhao Wu

**Affiliations:** Changchun Institute of Optics, Fine Mechanics and Physics, Chinese Academy of Sciences, Changchun 130033, China

**Keywords:** daytime detection, suppression of skylight background, shearing interference detection, double-grating structure, detection performance

## Abstract

To overcome the influence of the daytime skylight background on long-distance optical detection, a new type of shearing interference detection system was proposed to improve the detection performance of the traditional detection system for finding dark objects such as dim stars during the daytime. This article focuses on the basic principle and mathematical model as well as the simulation and experimental research of the new type of shearing interference detection system. The comparison of the detection performance between this new-type detection system and the traditional system is also carried out in this article. The experimental results show that the detection performance of the new type of shearing interference detection system is significantly better than that of the traditional system, and the image signal-to-noise ratio of this new-type system (about 13.2) is much higher than that of the best result of the traditional detection system (about 5.1).

## 1. Introduction

Optical detection techniques are widely used in remote sensing, searching, tracking, detecting, imaging, and other fields because of their advantages of short wavelength, high energy concentration, and high signal-to-noise ratio (SNR) detection. However, the skylight background is strong in the daytime, and it is easy to cover dim and weak targets, meaning daytime detection is always the weakness for the application of optical detection techniques (especially for ground-based equipment). To overcome the influence of the daytime skylight background, researchers have been exploring some methods to suppress the skylight background, mainly including the field of view (FOV) constraint [1], spectral filtering [2,3,4], polarization filtering [5,6], etc. However, these methods have their shortcomings. For the FOV control method, the drawbacks are that the FOV is too small and that the suppression of the strong skylight background in the FOV is poor. For the spectral filtering method, the drawback is that when the spectral characteristic of the target is similar to that of the skylight background, it is difficult to suppress the skylight background without lessening the target light. For the polarization filtering method, it is generally necessary to estimate in advance the discrepancy of the polarization characteristics between the target light and the skylight background, while the specific implementation is very complex because the polarization changes according to the orbital motion, the attitude change of the target, the solar movement, etc.

To avoid the above problems, the interference detection technique [7,8,9,10] has been proposed to improve the detection performance for finding dark stars. The interference detection technique uses the discrepancy of the far-field coherence between the target light and the skylight background to realize the separation of the two. It adopts a new light field characteristic independent of the traditional noise suppression methods, which do not conflict with the traditional ones. Therefore, the interference detection technique can be used alone to suppress the skylight background, or it can also be combined with traditional methods to achieve better suppression ability. Due to the shortcomings of the low interference contrast of target light and the low aperture utilization of the early interference detection technique based on electro-optic modulation devices [7], a shearing interference detection technique based on the double-grating structure [11,12,13,14] was proposed [10], which can significantly improve the interference contrast of target light and increase the aperture utilization. However, the published scheme of the shearing interference detection technique [10] uses a beam-shrinking telescope to reduce the interference fringe area and improve the fringe contrast. The beam-shrinking telescope realizes pupil transformation and reduces the large pupil to the small pupil. In essence, the published scheme only realizes pupil plane detection. It is difficult to distinguish the overlapping light field of different targets on the pupil plane. The image points of different targets can be transformed to the different positions on the image plane by the imaging lens (realizing the Fourier transform). Therefore, most of the traditional detection systems based on the principle of geometric optics utilize image plane detection. If shearing interference detection wants to achieve multi-target detection, it must also utilize image plane detection. The published shearing interference detection scheme [10] obviously cannot achieve image plane detection, and the conclusion of improving the detection SNR is also aimed at pupil plane detection.

To realize the application for multi-target scenes with the shearing interference detection system, this paper proposes the structural scheme of a new type of shearing interference detection system for image plane detection. The new structure includes the shearing interference device and the traditional detection system, which is the combination of the imaging lens and the array camera. This paper mainly includes the following contents. First, the basic principle and the mathematical models of the new type of shearing interference detection system are introduced briefly. Then, the simulation of the new system is shown in detail. Next, research on the detection performance of the new system is carried out by an experiment. Finally, the new system is compared with the traditional detection system to verify the superiority of the detection performance of the new scheme.

## 2. Basic Principle

**Figure 1:**
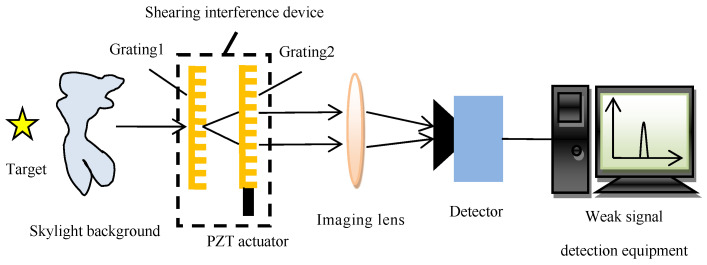
Conceptual diagram of the new type of shearing interference detection system.

The shearing interference detection technique takes advantage of the fact that the far-field coherence of the small-scale target is better than that of the large-scale skylight background, which is described by the Van Cittert–Zernike principle [15]. The conceptual diagram of the new type of shearing interference detection system is shown in Figure 1. The main difference in the structure scheme between this new-type detection system and the published shearing interference detection system is that the new system replaces the beam-shrinking telescope (meaning the pupil plane detection) with the imaging lens (meaning the image plane detection, which is the same as the application mode of the traditional optical detection system) so that the simultaneous detection of multiple targets in the field of view can be realized and the influence of stray lights in the pupil plane can also be suppressed. The small-scale target can form the interference field that changes periodically with time in the pupil plane by phase modulation, which is realized by transversely moving one of the gratings respective to the other, while the large-scale skylight background can hardly interfere. The interference field in the pupil plane is transformed into a small light spot (covering several pixels) in the image plane by the imaging lens, and the light spot intensity also changes periodically with time. The periodically changing spot intensity is converted into a periodic electrical signal by the array detector. The periodic target light signals can be extracted from the random skylight background noises by using the weak signal detection technique [16,17]. This technique can detect the sinusoidal signal with a very low SNR (even less than one). Therefore, in principle, the new type of shearing interference detection system can detect the target with a very low SNR to realize the detection of dim and weak targets in the strong skylight background.

Figure 1 shows that the main difference between the new type of shearing interference detection system (abbreviated as the new type of SIDS) and the traditional detection system is that there is one shearing interference device (abbreviated as the SID, boxed in Figure 1) that is composed of two gratings and one actuator placed in the pupil plane of the traditional detection system (often before the imaging lens as shown in Figure 1).

Referring to Figure 1, the working principle of the new type of SIDS is described below. The physical process of the target light forming the spot with periodic changes in light intensity on the detector is as follows. The target light first passes through the SID. Next, this light forms the interference field in the front plane of the imaging lens (i.e., the pupil plane). If a white screen is placed in the front plane of the imaging lens, the Moire fringe structure will be seen on this screen. The Moire fringes are generated by the rotation of grating 2 relative to grating 1 by an angle along the optical axis. When grating 2 is controlled to move along one direction in the grating plane vertical to the grating lines, the Moire fringes will move in one direction in the pupil plane. Conversely, the Moire fringes will move in the opposite direction. After passing through the imaging lens, the target light converges in the camera plane to form a small light spot whose intensity changes periodically with time. Due to the periodic variation of the light spot intensity and the random variation of the background light intensity, even if the background light intensity is much greater than the target light (that is, the background light completely covers the target light), the weak signal detection technique can also be used to detect the weak target light.

Based on the above principle, a brief introduction to the mathematical model of the new type of shearing interference detection system is given in the following section.

### 2.1. Interference Intensity Distribution of the Target Light in the Detector Plane

As mentioned earlier, the core principle of the shearing interference detection system is that after shearing interference, the target light can interfere while the background light does not, thereby distinguishing the target light from the background light based on their spatial coherence discrepancy. To facilitate the analysis of the working principle of the new type of shear interference detection system, the mathematical model introduced in this section assumes that the target light is a monochromatic plane wave, while the background light does not interfere. Therefore, in the mathematical model corresponding to the entire optical path from the target light incident on grating 1 to the formation of the target image in the camera plane, only the transmission and transformation of the target light are considered. The influence of the background light on the detection performance of the new type of SIDS will be considered in detail in the simulation with noises.

According to the wave optics theory and the angular spectrum transmission theory, the expression of the spatial spectrum of target light in the front plane of the imaging lens can be obtained:

(1) $$S_{lens}=F_{trans}(F_{trans}^{-1}(F_{trans}(E_{in}\cdot T_{1})\cdot H_{1})\cdot T_{2})\cdot H_{2}$$

In Equation (1): $S_{lens}$ is the spatial spectrum of target light before the imaging lens, and $E_{in}$ is the incident light field in the front plane of grating 1, which can be expressed as:

(2) $$E_{in}=A_{1}e^{i(\vec{k}\cdot \vec{r}+\phi_{0})}=A_{1}e^{i(ky\sin\theta +kz\cos\theta +\phi_{0})}$$

In Equation (2): $A_{1}$ is the amplitude of the incident light; $\vec{k}$ is the wave vector; $\vec{r}$ is the position vector; k=2π/λ is the size of the wave vector; λ is the wavelength; y and z are the coordinate values, respectively; θ is the angle between the wave vector and the z axis; and $\varphi_{0}$ is the initial phase.

$T_{1}$ is the amplitude transmittance of grating 1, which can be expressed as:

(3) $$T_{1}=\{\begin{array}{cc} 1 & y\in [nd,nd+d/2]\\ e^{i\pi } & y\in [nd+d/2,nd+d] \end{array}$$

In Equation (3): the external dimensions of grating 1 are ignored, and the phase difference between the convex and concave structures of grating 1 is assumed to be π. d is the grating period (or the grating constant), and n∈(−∞,+∞) is an integer.

$T_{2}$ is the amplitude transmittance of grating 2, which can be expressed as:

(4) $$T_{2}=T_{1}*\delta (y-\nu t)$$

In Equation (4): ∗ represents the convolution operator; δ(y) is the unit impulse function (when y=0, δ(y)=1, and others δ(y)=0); ν is the one-way moving speed of grating 2; and t represents the time.

$H_{1}$ is the amplitude transfer function between gratings 1 and 2, which can be expressed as:

(5) $$H_{1}=e^{ikz_{1}}e^{-i\pi \lambda z_{1}(f_{x}^{2}+f_{y}^{2})}$$

In Equation (5): $z_{1}$ is the distance between gratings 1 and 2, and $f_{x}=x/\lambda z_{1}$ and $f_{y}=y/\lambda z_{1}$ are the spatial frequencies, respectively.

$H_{2}$ is the amplitude transfer function between grating 2 and the imaging lens, which can be expressed as:

(6) $$H_{2}=e^{ikz_{2}}e^{-i\pi \lambda z_{2}(f_{x}^{\prime 2}+f_{y}^{\prime 2})}$$

In Equation (6): $z_{2}$ is the distance between grating 2 and the imaging lens, and $f_{x}^{\prime }=x/\lambda z_{2}$ and $f_{y}^{\prime }=x/\lambda z_{2}$ are the spatial frequencies, respectively.

$F_{trans}$ is the spatial-domain Fourier transform operator, and $F_{trans}^{-1}$ is the spatial-domain inverse Fourier transform operator.

Because the target light field in the detector plane is the spatial-domain Fourier transform of the light field before the imaging lens (the effect of the imaging lens is just the spatial-domain Fourier transform), the target light field ($E_{fri}$) in the detector plane is just the spatial spectrum of target light before the imaging lens ($S_{lens}$) with the assumption of no transmittance loss and no aperture limitation of the imaging lens.

The interference intensity distribution of the target light in the detector plane can be expressed as follows:

(7) $$I_{fri}=E_{fri}\cdot E_{fri}^{*}=S_{lens}\cdot S_{lens}^{*}$$

In Equation (7): $I_{fri}$ is the light intensity in the detector plane; $E_{fri}$ is the target light field in the detector plane; and the superscript * represents the complex conjugation.

So far, we have obtained the mathematical model of the formation process of the interference intensity distribution of the target light in the detector plane. The one-way motion of grating 2 results in the periodical change of the transmittance. By the later simulation, it can be shown that the above change will cause a periodic change in interference intensity distribution of the target light in the detector plane.

### 2.2. Image Reconstruction

**Figure 2:**
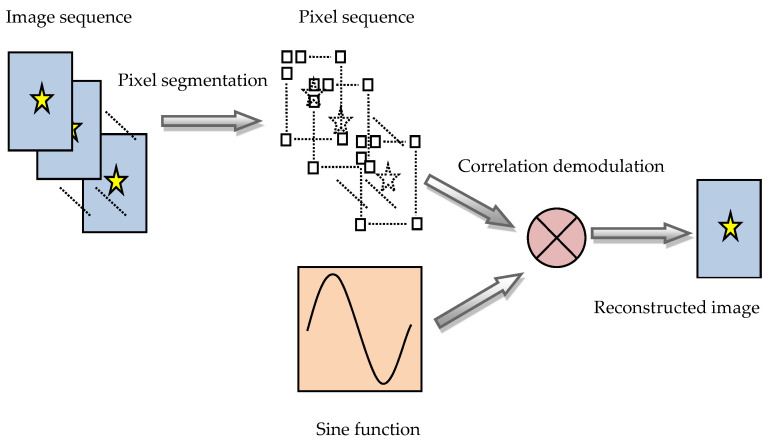
Image reconstruction process of the new type of SIDS.

By using the mathematical models of shearing interference and phase modulation described in Section 2.1, it is possible to generate an image sequence that includes the characteristics of the periodic variation over time of the target light intensity in the image plane. The pixels of the array detector corresponding to the positions of the target image can detect the periodic modulation characteristics of the target light intensity. The periodic modulation of light intensity can be considered as moving the target light intensity from zero frequency in the time-frequency domain (generally, the light intensity of a constant light-emitting body can be considered as direct current or zero-frequency modulation) to a higher frequency, thereby significantly separating it from the background light (mainly concentrated near zero frequency). Because the modulation is periodic and the frequency is known, such as the sinusoidal modulation, it is possible to divide the collected image sequence with multiple periods into many temporal data sequences corresponding to the pixels of the array detector and then demodulate each temporal data sequence separately. Finally, the demodulated values are reconstructed into an image based on the positions of the corresponding pixels in the array detector plane, which is the reconstructed image of the new type of SIDS. The above image reconstruction process is shown in Figure 2.

From the above description of the image reconstruction method, it can be seen that its core lies in the correlation demodulation of pixel data sequences. The periodic change in the target light generated by the phase modulation is close to the sinusoidal curve, so a sinusoidal function can be used for the correlation demodulation of pixel data sequences. The correlation demodulation relationship is:

(8) $$I_{pixel-rec}=I_{pixel-dec}⊗sin(2\pi \nu t)$$

In Equation (8): $I_{pixel-rec}$ is the pixel value after the correlation demodulation; $I_{pixel-dec}$ is the pixel data sequence; ⊗ is the correlation operator; sin(⋅) is the sinusoidal function; ν is the demodulation frequency equal to the modulation frequency; and t is the time

### 2.3. Image SNR Calculation

The calculation method of the signal-to-noise ratio of the new type of shear interference detection system introduced in this article can refer to that of the traditional detection system. The SNR of the traditional electro-optical detection system [18] can be expressed as:

(9) $$SNR_{trad}=\frac{S_{object}}{\sqrt{S_{object}+N_{sky}}}$$

In Equation (9): $S_{object}$ is the photoelectron number generated by the target light, and $N_{sky}$ is the photoelectron number generated by the skylight background. This equation applies to the situation with a known target light intensity and a known background light intensity, so the image SNR can be analyzed theoretically. Because the ultimate objective of the optical detection system is to obtain an image and the exact location of the target cannot be known when the signal-to-noise ratio is low, it is more practical to calculate the signal-to-noise ratio using the gray values of the image itself.

The calculation equation of the image SNR of the traditional detection system is [19,20]:

(10) $$SNR=S_{\max}/\sigma_{\mathrm{background}}$$

In Equation (10): $S_{\max}$ is the maximal value of the reconstructed image (the mean value of the background is removed), and σ is the standard deviation of the background outside the target.

The calculation equation of the image SNR of the new type of SIDS is the same as that of the traditional detection system.

## 3. Simulation

For the convenience of the analysis and the simulation, we make the following assumptions.

(I) It is assumed that the target light is a monochromatic plane wave and that its propagation in the free space and the optical elements can be described by the scalar wave optics;
(II) It is assumed that the size of grating 2 is large enough that the back-and-forth motion of grating 2 can be replaced by a one-way motion. It can cause periodic variation of the interference field intensity in the pupil plane and then cause periodic variation of the target image intensity in the image plane by passing through the imaging lens. The periodic variation of the target image intensity in the detector plane can produce a periodic signal, which is generally similar to the sinusoidal structure;
(III) It is assumed that the optimized shearing amount is used in the simulation, i.e., the skylight background cannot form the interference fringes, while the target can form high-contrast fringes. As the grating shearing interferometer can realize the continuous change of the shearing amount from zero to several centimeters, the optimized shearing amount can always be found by experiments;
(IV) It is assumed that the detector signal generated by the illumination of the skylight background is the Gaussian white noise and that this noise is far greater than other noises such as thermal noise, low-frequency noise, etc. Thus, there is only this noise and the photon fluctuation noise in the detection system. When the skylight background noise is larger than the amplitude of the target signal, the target signal will be submerged by the skylight background noises;
(V) It is assumed that the weak signal detection method used in our simulation is the correlation demodulation that is achieved by performing correlation operations between the periodic signal generated by the phase modulation and the sine function.

### 3.1. Simulation of the Shearing Interference and the Periodic Modulation of Phase Difference

**Figure 3:**
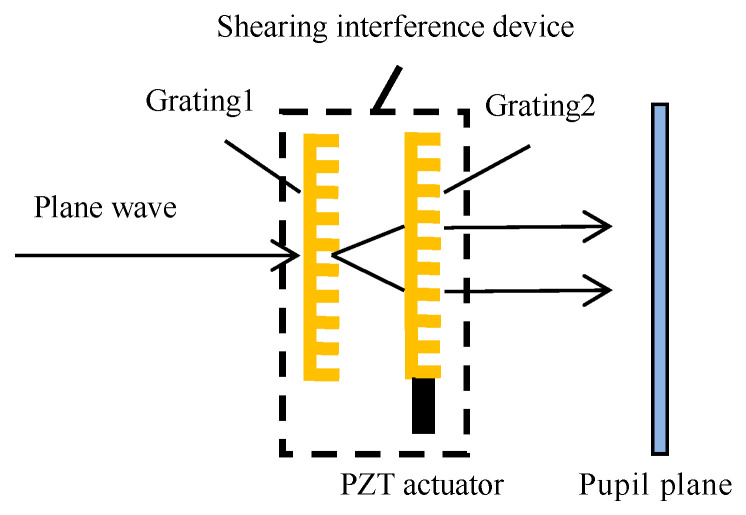
Optical path structure of the simulation of the shearing interference and the periodic phase modulation.

This section first simulates the shearing interference field generated by two gratings and the periodic modulation of the phase difference between the shearing beams formed by the relative motion between gratings. The simulation starts with a plane wave incident on grating 1 and then propagates through grating 2 to the pupil plane, forming a shearing interference field in this pupil plane. When grating 2 moves periodically back and forth with the grating period as its travel path, the phase difference between the two shearing beams will be periodically modulated. Through the subsequent simulation, it can be determined that the interference field intensity in the pupil plane will periodically change with time. The corresponding optical path structure of this simulation process is shown in Figure 3.

To obtain an intuitive explanation of the periodic change in the interference field caused by the grating motion, here, grating 2 is rotated at a small angle so that the two shearing beams form Moire fringes in the pupil plane. From the later simulation, it can be intuitively seen that when grating 2 periodically moves back and forth, the Moire fringes will periodically move. The simulation parameters are shown in Table 1.

**Figure 4:**
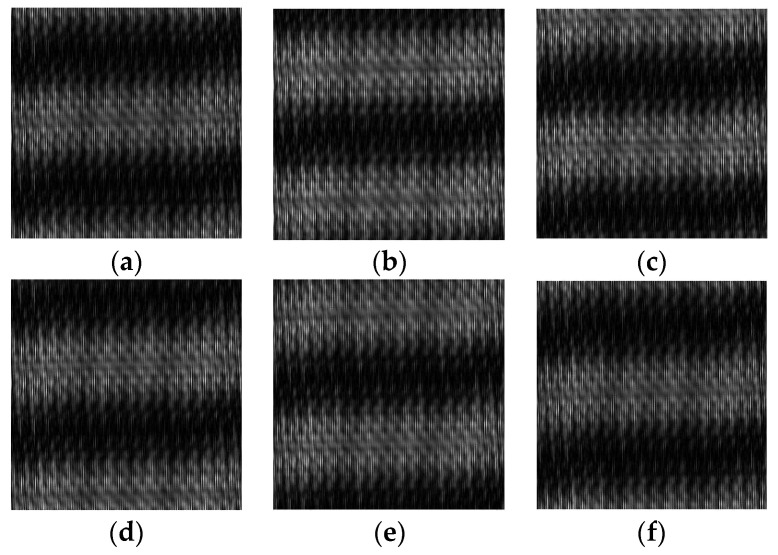
Simulation results of the shearing interference and the periodic phase modulation. (**a**–**f**) are the results when the Moire fringes move 0, 0.2, 0.4, 0.6, 0.8, and 1 fringe period, respectively.

The simulation results are shown in Figure 4. From the simulation results, it can be concluded that when grating 2 moves for one grating period in the direction perpendicular to the grating lines, the Moire fringes in the pupil plane also move for one period. It can be seen that when grating 2 periodically moves back and forth, the Moire fringes also periodically move back and forth, thereby achieving the periodic modulation of the shearing interference field.

### 3.2. Characteristics Simulation of the New Type of SIDS in the Image Plane

The research focus of this article is to transform the detection characteristics of the shearing interference detection system in the pupil plane into the detection characteristics in the image plane. The improvement effect of the shearing interference and phase modulation technique on the detection performance in the image plane is studied in detail. Starting from this section, the characteristics simulation of the new type of SIDS in the image plane will be carried out. This section mainly simulates the characteristics of the new type of SIDS in the image plane under ideal conditions (without background noises) and also gives the characteristics of the interference field in the pupil plane. The simulation parameters are shown in Table 2.

**Figure 5:**
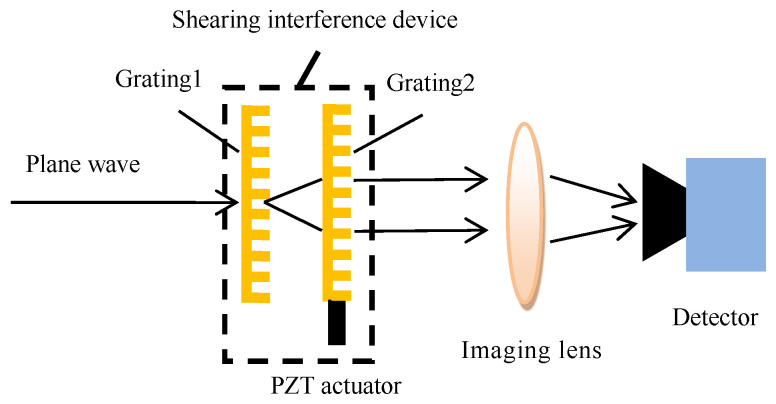
Optical path structure of the characteristics simulation of the new type of SIDS.

The simulation starts with a plane wave incident on grating 1, propagates through grating 2 to the imaging lens, and then enters the array detector plane through the imaging lens. The corresponding optical path structure of this simulation process is shown in Figure 5.

**Figure 6:**
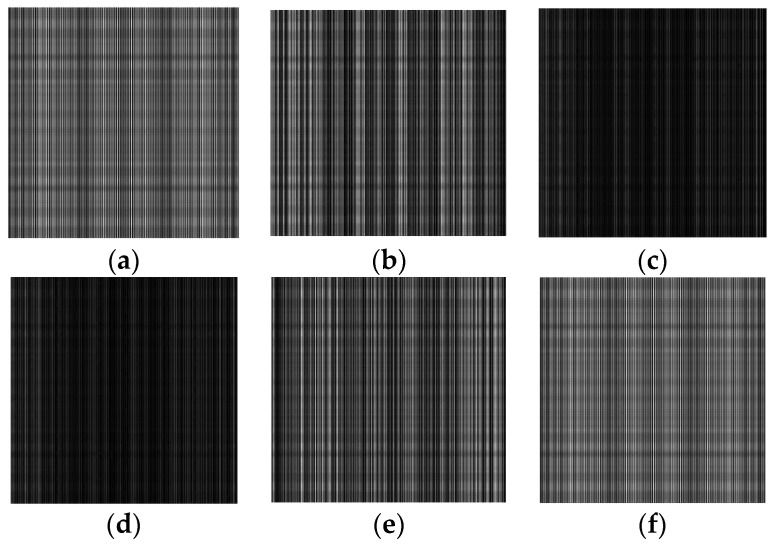
The simulation results of the interference field variation within a modulation period formed in the pupil plane. (**a**–**f**) are the results according to different moments of the modulation period (0, 0.2, 0.4, 0.6, 0.8, and 1 period, respectively).

The simulation of the interference field variation within a modulation period formed in the front plane of the imaging lens (i.e., the pupil plane) is shown in Figure 6. The interference field shown in Figure 6 is uniform. There is a fine fringe structure in Figure 6, resulting from the limited spatial sampling. The more spatial samples, the closer the light intensity distribution in the pupil plane is to the uniform distribution, but the simulation time significantly increases. From the changing trend of the interference field corresponding to different moments of the modulation period, it can be seen that when the rotation angle of the two gratings is 0°, the entire interference field in the pupil plane undergoes periodic changes simultaneously.

**Figure 7:**
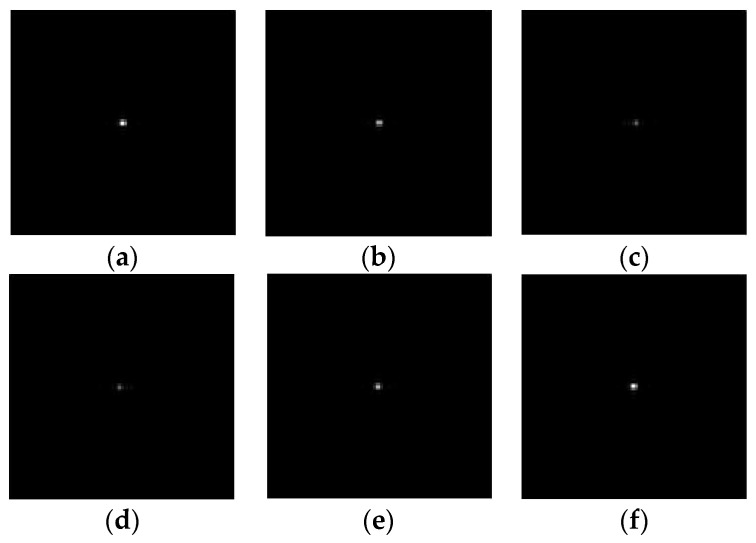
The simulation results of the variation of the target image brightness within a modulation period formed in the image plane. (**a**–**f**) are the results according to different moments of the modulation period (0, 0.2, 0.4, 0.6, 0.8, and 1 period, respectively).

The simulation of the interference field variation within a modulation period formed in the image plane of the imaging lens is shown in Figure 7. The light spot shown in Figure 7 is the target image passing through the imaging lens. From the variation trend of the spot brightness corresponding to different moments in the modulation period, it can be seen that the light intensity of the target image is modulated, causing periodic changes between brightness and darkness.

**Figure 8:**
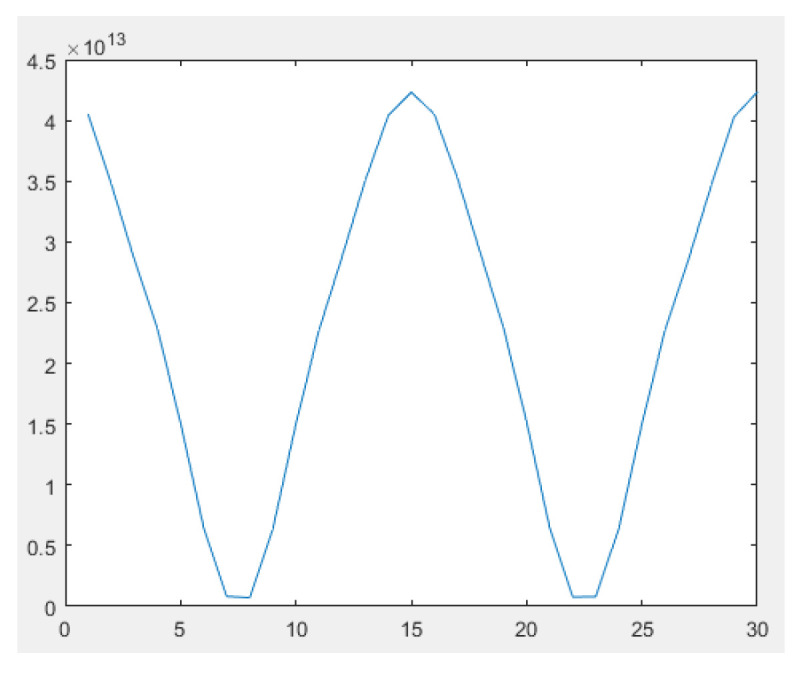
Curve of the gray values of the target image spot during two modulation cycles.

The pixel corresponding to the highlight position of the target image generated by the simulation of the new type of SIDS was chosen, the corresponding gray values of the pixel during two modulation cycles were obtained, and then the curve of the gray values of this pixel was drawn in Figure 8. As can be seen from Figure 8, after the target light undergoes the shearing interference and the periodic phase modulation, the image spot intensity formed in the image plane undergoes a periodic change similar to the sinusoidal curve. As the background light does not interfere in theory, it does not produce periodic changes in the light intensity in the image plane. The discrepancy between the target light and the background light can be generated by the shearing interference and the phase modulation, and then the dim target can be extracted from the scene covered by background noises utilizing the weak signal demodulation technique. The performance simulation of the new type of SIDS with background noises will be described in the next section.

### 3.3. Simulation of Detection Performance of the New Type of SIDS with Background Noises

The skylight background is equivalent to a large target that fills the entire field of view, with a small coherent region in the far field (about the order of micrometers for the visible light). When the shearing amount between two gratings (i.e., the center distance between the two shearing beams) is of the order of mm, the two shearing beams generated by the background light theoretically do not interfere with each other. After passing through the SID and the imaging lens, the background light forms an approximate uniformly random distribution of the light intensity in the image plane.

In the simulation, it is assumed that the background light is a Gaussian random variable with a mean value of 10 and a standard deviation of 1, while the brightest light intensity value of the target is 1; thus, the target’s SNR is 1. The simulation parameters are shown in Table 3.

**Figure 9:**
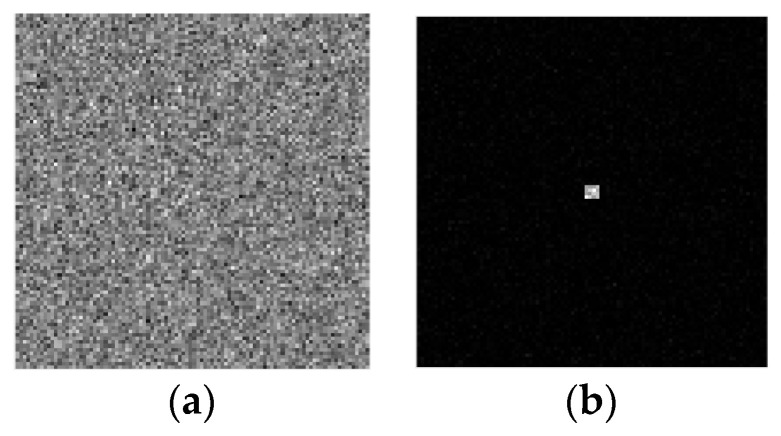
Single-frame image and final reconstructed image of the new type of SIDS. (**a**) single-frame image in the modulated target image sequence and (**b**) the reconstructed image of the new type of SIDS (SNR = 16.9312).

The simulation generated a noisy modulated target image sequence with 1500 frames (corresponding to 100 modulation cycles). The first brightest target image in the modulated target image sequence is shown in Figure 9a. As can be seen from Figure 9a, due to the extremely low signal-to-noise ratio (about 1), the target cannot be found in a single frame image. The image reconstructed from the above image sequence by using the interference detection technique is shown in Figure 9b. As can be seen from Figure 9b, the interference detection technique can effectively suppress the background noises and generate the image with a very dark background, making it easy to detect the dim target (SNR = 16.9312).

**Figure 10:**
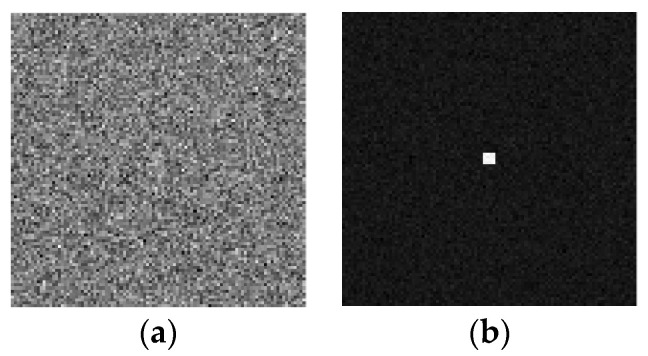
Single-frame image and the multi-frame average image of the traditional detection system. (**a**) single-frame image and (**b**) multi-frame average image (SNR = 9.1732).

To compare the detection performance of the new type of SIDS and the traditional detection system, the following is the simulation of the detection performance of the traditional detection system. The simulation uses the same amount of data as the new type of SIDS (image sequence with 1500 frames). Unlike the new type of SIDS, the target in this image sequence is not modulated (i.e., the target brightness value is constant). The single-frame image and the multi-frame average image of the stationary target in the image sequence are shown in (a) and (b) of Figure 10, respectively.

By comparing the interference detection image and the multi-frame average image, it was found that the target local brightness of the interference detection image was lower than the multi-frame average image, while the background noises of the multi-frame average image were larger than the interference detection image. The SNR of the interference detection image (16.9312) was greater than the SNR of the multi-frame average image (9.1732).

Theoretically, for completely random noises, the final effect of the multi-frame averaging method and the modulation and demodulation method is the same with the same frame number (i.e., the same amount of data) of the image sequence. However, in practical applications, there are always structural noises (deterministic noises), and the effect of multi-frame averaging methods will not be as good as the modulation and demodulation methods. In other words, in practical application scenarios where there are some structural noises, the detection performance of the new type of shearing interference detection system described in this article will be superior to the traditional detection system. Subsequent indoor experiments will confirm this view.

## 4. Experimental Setups and Methods

**Figure 11:**
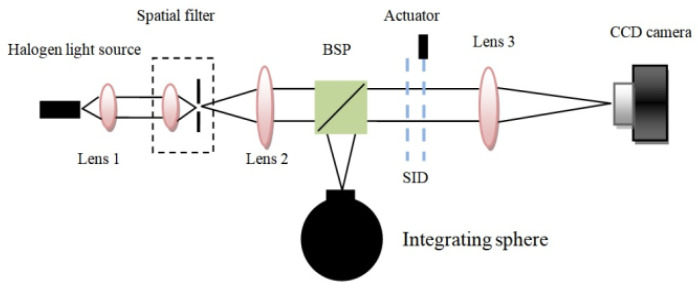
Experimental scheme of the new type of SIDS.

**Figure 12:**
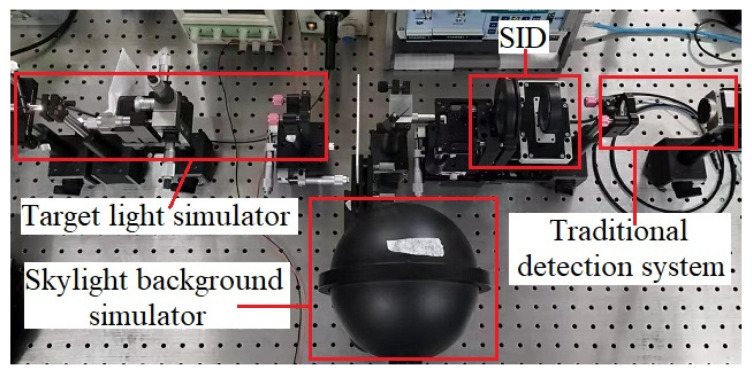
Experimental setup of the new type of SIDS.

The experimental scheme of the new type of shearing interference detection system is shown in Figure 11. In Figure 11, the optical layout mainly includes four parts, which are the target light simulator, the skylight background simulator, the SID, and the traditional detection system. The white light point target simulator is composed of the halogen lamp (Daheng Optics, Beijing, China, GCI-060101), lens 1 (Daheng Optics, GCL-010104), and the spatial filter (Newport Model 900) to generate the target light of which the diameter of the far-field coherent area is nearly equal to the diameter of the system optical path. Lens 2 (Daheng Optics GCL-010650) collimates the target light into the plane wave to simulate the target at a far distance. The integrating sphere will produce nearly uniform stray light to simulate the characteristics of the skylight background. The target light and the stray light are combined by the beam split prism (BSP, Daheng Optics GCC-403102) to simulate a situation in which the target light is covered by the skylight background. The SID composed of two gratings and one actuator can split the combined lights from the BSP into two transverse shearing beams. The gratings of the SID are two identical Ronchi phase gratings (HOMO GS-2-532-50U). One side of the second grating is connected with the actuator (Core Morrow, Harbin, China, P66) to realize the linear periodic back-and-forth motion. Because the transverse displacement of the two shearing beams is far less than the far-field coherent region of the target light, the two shearing beams from the target light can form a nearly ideal interference field. The two shearing beams are parallel plane waves, so the intensity distribution of the interference field is uniform. When the actuator moves back and forth, the intensity of the interference field will change periodically. After the SID, there is the traditional detection system, which is composed of lens 3 and the CCD camera. Lens 3 (Daheng Optics GCL-010111) focuses the interference field energy to the small size spot on the CCD camera (Basler piA1600-35gm). The CCD camera can receive serial image frames, which include the modulated information of the target light. By the signal demodulation method (such as the Fourier transform of time series data for each pixel in the camera), the reconstructed image of the shearing interference detection system can be obtained. The experimental setups of the new type of detection system are shown in Figure 12.

When the periodic modulation of the optical path difference between two shearing beams is introduced by the back-and-forth movements of the actuator at a low frequency (such as 1 Hz), the light intensity on the pixels of the detector will change periodically with time. Then, the CCD camera can obtain a sequence of modulated image frames (2000 frames here), which reflects the trend of the received light intensity on the CCD camera changing with time. By segmenting the image frame sequence according to pixels, the changing trend over time of the gray value of any pixel in the image can be obtained. The time series of gray values of any pixel in the image will be Fourier transformed, and then the spectrum value corresponding to the modulation frequency (1 Hz here) will be chosen as the demodulation value of this pixel. The demodulated pixels are recombined into an image according to their positions on the CCD camera. This final image is just the reconstructed image of the new type of detection system.

When the target light is covered by the skylight background, the total light intensity on the detector can be expressed as:

(11) $$I_{total}=I_{fri}+I_{bg}$$

In Equation (11): $I_{total}$ and $I_{bg}$ are the total light intensity and the skylight background intensity on the detector, respectively. Due to the random change in the skylight background with time, its contribution to the modulation frequency is very little. Therefore, the power spectrum analysis can distinguish the target light from the skylight background noise when the SNR is very low. The following experimental results will verify that the new type of detection system can effectively suppress the influence of the stray light (simulating the skylight background) to effectively extract the target with a low SNR.

When the SID is removed from the optical layout as shown in Figure 11, it becomes just the experiment scheme of the traditional detection system. To accurately compare the difference in the detection performance between the new type of detection system and the traditional one, the traditional detection system also uses image data of 2000 frames to reconstruct the image. One of the most commonly used and effective methods of reconstructing the final image in traditional detection systems is the multi-frame averaging method (abbreviated as the MFAM). The improvement of the image SNR by this method is proportional to $\sqrt{N}$ (provided that there is only random noise in the image), and N is the number of frames. Here, this method is used to reconstruct the final image. By experiment, it is found that there are some structural noises (almost unchanged with time) in the final image after multi-frame averaging, which cannot be eliminated by this method. Therefore, we use the background fitting subtraction method (abbreviated as the BFSM) by which the background of the image is fitted first and then subtracted from the image to eliminate the influence of structural noises. To conveniently compare the detection performance of different methods (multi-frame averaging and background fitting subtraction), the intensity figures and the mesh figures are both applied.

## 5. Results and Discussion

### 5.1. Traditional Detection System

**Figure 13:**
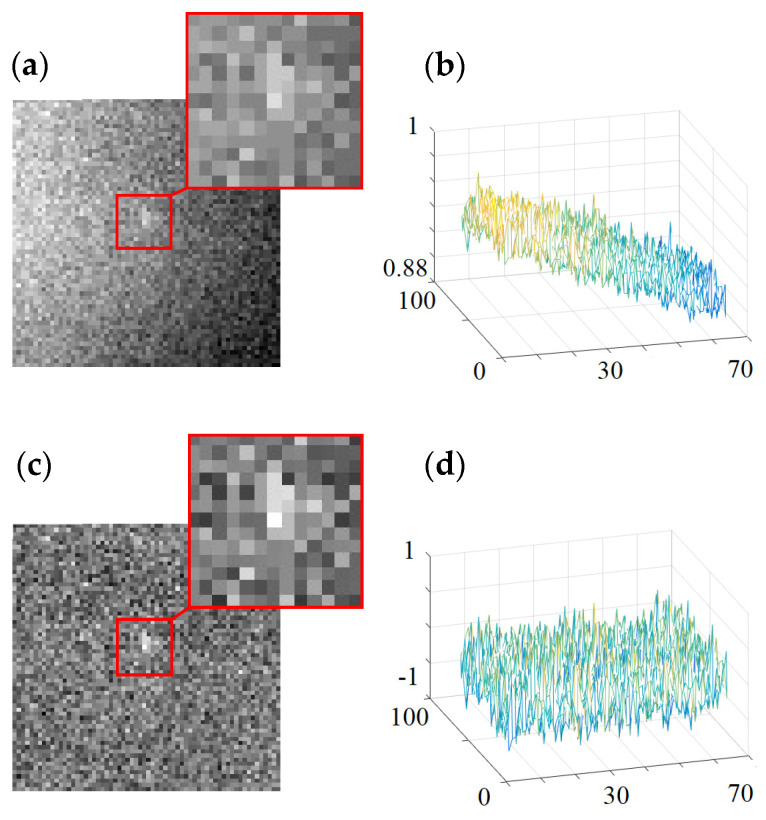
Single-frame images of the traditional detection system: (**a**) intensity figure of raw data (SNR = 2.9), (**b**) mesh figure of raw data, (**c**) intensity figure with background fitting subtraction (SNR = 4.4), and (**d**) mesh figure with background fitting subtraction.

The single-frame raw image and the single-frame image with background fitting subtraction are shown in Figure 13. It can be seen in Figure 13 that there is a slope structure (structural noise) in the background of Figure 13b, which can be eliminated by the background fitting subtraction method (almost no slope structure in Figure 13d). The image SNR in Figure 13c (about 4.4) is larger than that in Figure 13a (about 2.9), which illustrates that structural noise is the major noise in raw images that can be eliminated by the background fitting subtraction method.

**Figure 14:**
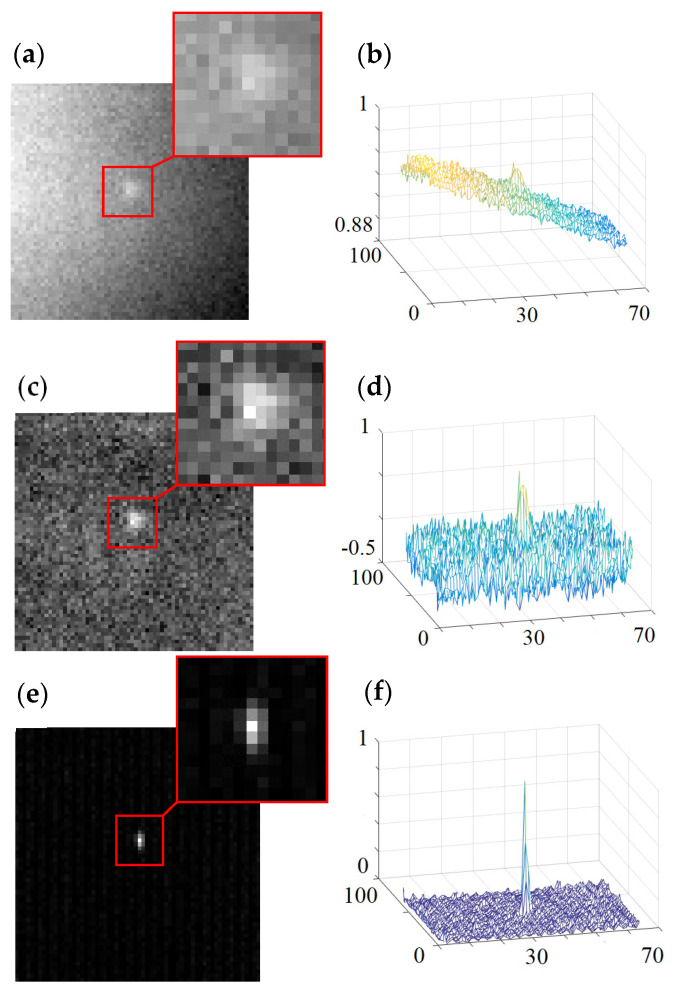
Comparison between the traditional detection system and the new type of SIDS: (**a**) intensity figure (SNR = 2.9) and (**b**) mesh figure from the traditional detection system by the MFAM, (**c**) intensity figure (SNR = 5.1) and (**d**) mesh figure from the traditional detection system by the CM, and (**e**) intensity figure (SNR = 13.2) and (**f**) mesh figure from the new type of SIDS.

The reconstructed images of the traditional detection system by the multi-frame averaging method and by the combined method (abbreviated as the CM) with multi-frame averaging and background fitting subtraction are shown in Figure 14. According to the SNR equation and the reconstructed images, the image SNRs by the multi-frame averaging method and by the combined method are about 2.9 and 5.1, respectively. The mesh figure in Figure 14b is similar to the mesh figure in Figure 13b, which illustrates that the structural noise (slope structure) cannot be eliminated by multi-frame averaging. The image SNR in Figure 14a (about 2.9) is the same as that in Figure 13a (about 2.9), which also illustrates that structural noise is the major noise in raw images that cannot be eliminated by multi-frame averaging.

### 5.2. New-Type Shearing Interference Detection System

These reconstructed images of the new type of shearing interference detection system are shown in Figure 14e,f under the same condition of the target and background brightness as that of the traditional detection system. The calculation equation of the image SNR is the same as that of the traditional detection system, and the result is about 13.2.

The SNR results of reconstructed images from the traditional detection system and the shear interference detection system are summarized in Table 4. From Table 4, it can be seen that the SNR of the new type of SIDS is significantly better than that of the traditional detection system with the same amount of data.

By the comparison of experimental results (shown in Figure 14 and Table 4), it can be concluded that the reconstructed image of the new type of detection system has a much higher image SNR, and visually, the background noise in this image is very weak (almost disappeared), and the target in this image is visible. This shows that by introducing the shearing interference method and the modulation and demodulation method and utilizing imaging optics instead of the beam-shrinking telescope, the new type of shearing interference detection system can make full use of the target light information and can effectively suppress background light noises so that its detection performance on the image plane is significantly better than that of the traditional detection system.

## 6. Conclusions

The performance of the new type of shearing interference detection system was studied by a simulation with the monochromatic plane wave approximation and an experiment with the white light (wide spectrum) point target covered by the wide spectrum stray lights, which is a good approximate to the real daytime scene. The experimental results show that the new type of detection system can effectively suppress the influence of stray lights and can extract a lower SNR target on the image plane from strong noises more effectively than the traditional detection methods. From the perspective of hardware, the new type of detection system just has one more shearing interference device than the traditional detection system, so we can easily add this device to the existing traditional detection system and then use the image reconstruction method of the shearing interference detection technique to significantly improve the daytime detection ability of the traditional detection system.

## Figures and Tables

**Table 1:** Simulation parameters of the shearing interference and the periodic phase modulation.

Simulation Parameters	Type or Value
Incident light	Monochromatic plane wave
Wavelength	0.5 μm
Grating period	50 μm
Sampling points per cycle	30
Grating size	2.5 mm × 2.5 mm
Rotation angle between gratings 1 and 2	3°
Distance between gratings 1 and 2	4 mm
Distance between grating 2 and the pupil plane	4 mm
Sampling points in pupil plane	500 × 500

**Table 2:** Simulation parameters of the new type of SIDS.

Simulation Parameters	Type or Value
Incident light	Monochromatic plane wave
Wavelength	0.5 μm
Grating period	50 μm
Sampling points per cycle	30
Grating size	2.5 mm × 2.5 mm
Rotation angle between gratings 1 and 2	0°
Distance between gratings 1 and 2	4 mm
Distance between grating 2 and the pupil plane	4 mm
Sampling points in the pupil plane	500 × 500
Sampling points in the image plane	100
Sampling points per modulation period	15

**Table 3:** Simulation parameters of the new type of SIDS with background noises.

Simulation Parameters	Type or Value
Incident light	Monochromatic plane wave
Wavelength	0.5 μm
Grating period	50 μm
Sampling points per cycle	30
Grating size	2.5 mm × 2.5 mm
Rotation angle between gratings 1 and 2	0°
Distance between gratings 1 and 2	4 mm
Distance between grating 2 and the pupil plane	4 mm
Sampling points in the pupil plane	500 × 500
Sampling points in the image plane	100
Sampling points per modulation period	15
Target SNR	1
Modulation cycles with noises	100

**Table 4:** Reconstructed SNRs of the traditional detection system and the new type of SIDS.

SNR of Single Frame	SNR of Single Frame with BFS	SNR of Multi-Frames with MFA	SNR of Multi-Frames with CM	SNR of the New Type of SIDS
2.9	4.4	2.9	5.1	13.2

## Data Availability

The data presented in this study are available on request from the corresponding author. The data are not publicly available due to technological security.
